# Relationship between Adiponectin Level, Insulin Sensitivity, and Metabolic Syndrome in Type 1 Diabetic Patients

**DOI:** 10.1155/2013/535906

**Published:** 2013-07-17

**Authors:** Kristina Blaslov, Tomislav Bulum, Karin Zibar, Lea Duvnjak

**Affiliations:** Vuk Vrhovac Clinic for Diabetes, Endocrinology and Metabolic Diseases, University Hospital Merkur, School of Medicine, University of Zagreb, Zagreb, Croatia

## Abstract

*Objective*. Adiponectin is known to be decreased in insulin resistance (IR) and metabolic syndrome (MS) which can be present in patients with type 1 diabetes mellitus (T1DM). The aim of this study was to evaluate the relationship between adiponectin level, MS, and insulin sensitivity in T1DM. *Research Design and Methods*. The study included 77 T1DM patients divided into two groups based on the total plasma adiponectin median value. Insulin sensitivity was calculated with the equation for eGDR, and MS was defined according to International Diabetes Federation criteria. *Results*. Patients with higher adiponectin level (*n* = 39) had significantly lower waist circumference (*P* < 0.002), fasting venous glucose levels (*P* < 0.001), higher HDL3-cholesterol (*P* = 0.011), and eGDR (*P* = 0.003) in comparison to the group with lower adiponectin who showed higher prevalence of MS (*P* = 0.045). eGDR increased for 1.09 mg/kg^−1^ min^−1^ by each increase of 1 **µ**g/mL total fasting plasma adiponectin (*P* = 0.003). In the logistic regression model, adiponectin was inversely associated with the presence of MS (*P* = 0.014). *Conclusion*. Higher adiponectin concentration is associated with lower prevalence of MS in T1DM. Whether higher adiponectin concentration has a protective role in the development of the MS in T1DM needs to be clarified in future follow-up studies.

## 1. Introduction

White adipose tissue is a major site of energy storage and is important for energy homeostasis. It has been increasingly recognised as an important endocrine organ that secretes a number of biologically active “adipokines” like tumor necrosis factor-*α*, plasminogen activator inhibitor-1, leptin, resistin, angiotensinogen, and adiponectin [1]. Some of these adipokines are associated with obesity-related conditions. Adiponectin has attracted much attention because of its antidiabetic and antiatherogenic effect, and it might become a novel therapeutic tool for type 2 diabetes mellitus (T2DM) and metabolic syndrome (MS) [2, 3]. According to several studies, plasma concentration of adiponectin is reduced in human obesity, particularly visceral, and negatively correlated with insulin resistance (IR) [4, 5]. Additionally, hypoadiponectinemia is independently associated with the MS, surrogate marker of IR, and significantly related to T2DM development [5–7]. MS represents an important risk factor for mortality and micro- and macrovascular complications development both in patients with type 1 diabetes mellitus (T1DM) and T2DM [8]. Adiponectin was found to accumulate in damaged vascular walls and beneficially modulate the endothelial inflammatory response to vascular injury as it possesses antiinflammatory and antiatherogenic properties [9]. Consequently, normal adiponectin concentrations or even induction of elevated concentrations are considered to be beneficial. However, increased adiponectin concentrations have been found to be associated with an increased cardiovascular mortality in T1DM [10, 11], but in cross-sectional data, the elevation in adiponectin levels has been hypothesised as a compensatory response in T1DM patients who have microvascular complications [10]. In addition, there are a growing number of patients with T1DM and MS who appear to be at increased risk of cardiovascular mortality and development of diabetes related complications, a greater need for higher insulin doses, and multifactorial intervention, thus, more aggressive treatment [12, 13]. Therefore, the purpose of this study was (1) to determine the relationship between total plasma adiponectin concentrations and parameters associated with the MS: body mass index (BMI), waist circumference, lipid profile, blood pressure levels, fasting glucose levels, and insulin sensitivity, (2) to determine the relationship between adiponectin concentrations and MS prevalence, and (3) to evaluate the possible pathophysiological role of adiponectin in MS development in T1DM patients defined according to International Diabetes Federation (IDF) criteria. 

## 2. Subjects, Materials, and Methods

This cross-sectional study was undertaken at the University Clinic for Diabetes, Endocrinology, and Metabolic Diseases Vuk Vrhovac, Zagreb, Croatia. The study population consisted of 77 T1DM patients coming for their comprehensive annual review with following characteristics: age of 18–65 years, minimum duration of type 1 diabetes of 1 year, no medical history of cardiovascular diseases or electrocardiogram (ECG) evidence of ischemic heart disease, absence of any systemic disease, and absence of any infections in the previous month. T1DM was defined as age at onset of diabetes younger than 35 years, positive autoantibodies, and time to definite insulin therapy less than a year. Patients were excluded from the study if they had taken any of the following: thyroid hormone therapy, medications that might affect glucose metabolism and insulin sensitivity such as glucocorticoids, oral contraceptives, and patients taking oral glucose-lowering medication. They could be using antihypertensive or lipid lowering drugs (i.e., statins: atorvastatin and simvastatin). Acute and chronic inflammation was excluded on the basis of medical history, physical examination, and routine laboratory tests, including measurement of temperature and urinalysis.

All subjects were studied in the morning between 08:00 am and 09:30 am after an overnight fast. Waist circumference was measured on bare skin as the narrowest circumference between the 10th rib and the iliac crest with tailor meter. Weight was measured by the physician using a balanced-beam scale with light clothing without shoes and expressed in kilograms (kg). Height was measured using a wall-mounted stadiometer and expressed in centimetres (cm). Body mass index (BMI) was calculated as weight in kilograms divided by the square of height in meters (kg/m^2^). Blood pressure was measured in the sitting position with a mercury sphygmomanometer after a resting period of 10 minutes and expressed in mmHg. Fasting venous blood samples were collected for the determination of biochemistry panel, lipid profile status, HbA1c, liver biochemistry, and adiponectin. HbA1c was measured spectrophotometrically by turbidimetric immunoinhibition (Olympus AU600, Beckman Coulter, USA). Glucose, cholesterol, and triglycerides in serum were measured by an enzymatic colorimetric method. Total fasting adiponectin was measured using BioVendor's Human ELISA (sandwich) commercial kit for research use only. Insulin sensitivity was calculated using the equation derived from euglycemic-hyperinsulinemic clamp studies estimated glucose disposal rate (eGDR): 24.31 − 12.2*X*(WHR) − 3.29*X*(AHT) − 0.57*X*(HbA1c), where the units are mg/kg^−1^min^−1^, WHR indicates the waist to hip ratio, AHT indicates blood pressure and is expressed as 0—no, 1—yes. Those on blood pressure medications or with blood pressure >140/90 mmHg were considered to have hypertension [14]. This equation was derived from a substudy of 24 EDC (Epidemiology of Diabetes Complications) participants (12 men and 12 women drawn from low, middle, and high age-specific tertiles of insulin resistance risk factors in order to represent the spectrum of insulin resistance) who underwent euglycemic-hyperinsulinemic clamp studies [15]. It should be emphasized that lower eGDR levels indicate greater insulin resistance. According to the IDF guidelines, participants were defined as having MS if they had central obesity defined as waist circumference with ethnicity specific values (>80 cm in female and 94 Cm in male population) plus any two of the following four factors: raised TG concentration: >150 mg/dL (1.7 mmol/L), specific treatment for this lipid abnormality, reduced HDL cholesterol: <40 mg/dL (1.03 mmol/L) in males and <50 mg/dL (1.29 mmol/L) in females, specific treatment for this lipid abnormality, raised blood pressure: systolic BP >130 or diastolic BP >85 mmHg, treatment of previously diagnosed hypertension, raised fasting plasma glucose (FPG): >100 mg/dL (5.6 mmol/L), or previously diagnosed type 2 diabetes [16].

The study protocol complies with the Declaration of Helsinki as well as with local institutional guidelines and was approved by the local ethics committees. Written informed consent was obtained from all participants.

Baseline data were reported using descriptive statistics. Normality of distribution for continuous variables was analysed using Shapiro-Wilk test. Normally distributed variables were described with mean and standard deviation (SD), while variables that were not normally distributed were described with median, minimum, and maximum. The nominal variables were reported with absolute numbers and/or percentages. Differences between groups were examined, depending on the nature of the data, using parametric (*t*-test) or nonparametric tests (Mann-Whitney). Correlations between parameters of adiponectin with anthropometric and metabolic variables were determined using Pearson's or Spearman's correlation coefficient. The association between total fasting adiponectin concentrations and eGDR value was further evaluated in univariate linear regression, and in order to evaluate the association of total fasting adiponectin concentration with MS presence, we constructed multivariable binary logistic regression models to assess whether circulating total adiponectin concentration was independently associated with MS. Adjustments were performed for age, gender, disease duration, and the use of statins since they are shown to affect adiponectin secretion [17]. Level of statistical significance was chosen to be 0.05. Statistical analysis was performed by Statistical Package for the Social Sciences (SPSS) ver. 17.0 for Windows.

## 3. Results

The characteristics of the study subjects are listed in Table 1. The average age was approximately 46 years, median BMI was 25 kg/m^2^, and 61% of patients were males with median diabetes duration of 21 years. Forty-five (58.4%) were using antihypertensive therapy and 33 (42.9%) hypolipedemic medications. Patients were divided according to the median adiponectin value (14.1 (3.4–31.4) *μ*g/mL). The group of patients (*n* = 38) with lower adiponectin concentrations (<14.1 *μ*g/mL) had significantly shorter disease duration, higher weight, BMI, waist circumference, GGT, and fasting venous glucose levels (8.45 versus 6.80 mmol/L, *P* < 0.001), while lower HDL3-cholesterol and eGDR compared to the group (*n* = 39) with higher adiponectin concentrations (>14.1 *μ*g/mL) (Table 2). No significant difference was found in the use of antihypertensive (60.5% versus 56.4%) or hypolipedemic (39.5% versus 46.2%) therapy between the two groups. Associations of adiponectin concentrations with anthropometric and metabolic variables are presented in Table 3. Disease duration, total HDL and HDL3 cholesterol showed significantly positive correlation with adiponectin concentrations; weight, BMI, waist circumference, systolic blood pressure, and eGDR showed significantly negative correlation with adiponectin concentrations. Between all study patients, 26 (33.8%) met the metabolic syndrome criteria.

In linear regression model, the unadjusted eGDR increased for 1.09 mg/kg^−1^ min^−1^ by each increase of 1 *μ*g/mL total plasma adiponectin (CI (1.03–1.16), *P* = 0.003). In the multivariate binary logistic regression, total fasting plasma adiponectin was inversely associated with presence of MS after adjustment for age, sex, disease duration, and the statin use (Table 4). The other variables included in the final model were not shown to be significant predictors of the MS presence (age OR = 0.977, *P* = 0.459; gender OR = 0.639, *P* = 0.418; disease duration OR = 1.022, *P* = 0.452; and the use of statins OR = 0.886, *P* = 0.140).

## 4. Discussion

Although pathophysiology of T1DM is defined with an autoimmune destruction of pancreatic *β* cells, increased IR has an important role in the development of so called “double diabetes” and greater risk for micro- and macrovascular complications [18]. MS and increased IR can be found in about 30%–40 % of T1DM patients depending on the used MS definition [19–21]. The association of lower adiponectin concentrations with the risk of MS development was previously shown in both healthy population and T2DM patients [5–7, 22]. 

Our results show that patients with higher plasma adiponectin concentrations had significantly lower BMI and waist circumference and higher HDL 3 cholesterol. The levels of systolic blood pressure, fasting plasma glucose, and triglyceride were also lower although they did not reach statistical significance. Additionally, the group with higher plasma adiponectin showed higher eGDR levels indicating higher insulin sensitivity which is in accordance with Pereira et al. (2012) [23] who reported that both total and high molecular weight (HMW) adiponectin positively correlate with insulin sensitivity assessed by hyperinsulinemic-euglycemic clamp in T1DM patients. Pham et al. (2013) [24] also investigated the association of total plasma adiponectin, anthropometric and metabolic parameters and found an inverse association of total plasma adiponectin and fasting glucose levels as well as BMI. About 35%–40% of the observed patient population met MS criteria, which is in accordance with the literature data [12, 18]. In the multivariate logistic regression after adjustment for age, gender, disease duration, and the use of statins, adiponectin concentration was significantly inversely associated with the prevalence of the MS. Those data suggest that higher adiponectin concentrations might play an important protective role in the development of MS in T1DM patients. 

Adiponectin has been shown to have insulin-sensitizing effects through activation of AMPK in the peripheral tissues [25]. These effects include stimulation of fatty acid oxidation and glucose uptake in skeletal muscle and suppression of glucose production in the liver [26]. Several experimental studies suggested that administration of adiponectin ameliorates IR in lipoatrophic and T2DM while it decreases plasma glucose levels in healthy mice [26, 27]. For those reasons, adiponectin is considered to be one of the major insulin-sensitizing hormones strongly associated with IR related disorders. Additionally, regarding the glucose metabolism, Berg et al. (2001) have shown that the administration of adiponectin lowers circulating glucose levels without stimulating insulin secretion in both healthy and diabetic mice [28], and the data from two independent studies performed by Kriketos et al. (2004) as well as Kim et al. (2007) show that exercise induced increased insulin sensitivity in humans could be due to elevation in total plasma adiponectin concentration in humans [29, 30].

Moreover, our finding of lower LDL cholesterol and TG as well as higher total HDL, HDL2, and HDL3 cholesterol in the group with higher plasma adiponectin is in accordance with a growing body of literature data suggesting that adiponectin has a direct effect on the regulation of lipid metabolism. Decreased adiponectin concentrations have been linked to higher LDL cholesterol and TG concentrations probably due to adiponectin directly affecting lipoprotein lipase [31, 32]. Data from two large cross-sectional studies indicate that circulating adiponectin concentrations are negatively correlated with triglyceride concentrations and strongly positively correlated with plasma HDL concentrations [32, 33]. There is even an assumption based on several study reports that treatment with some of the most used lipid lowering drugs such as statins and fibrates increases plasma adiponectin concentration which might contribute to their beneficial effect in LDL and triglyceride lowering effect [34]. The LDL cholesterol particle is the major cholesterol transporter shown to be a strong independent risk factor for atherosclerotic events associated with MS [35]. Regarding HDL cholesterol and the process of atherogenesis, it has been shown that both quantitative and qualitative particle alterations play an important role [36]. In particular, circulating HDL2 cholesterol was found to protect from atherosclerosis [37, 38], while we previously observed that HDL3 cholesterol might protect from progression of renal disease in type 1 diabetes [38]. That might be of particular importance in this population as T1DM represents a chronic condition with an increased cardiovascular mortality [10, 11]. Beyond observed, adiponectin might exert a protective effect on atherogenesis by preventing endothelial damage and decreasing blood pressure levels. 

There are several studies suggesting the inverse correlation of circulating adiponectin concentrations and elevated arterial blood pressure levels in healthy subjects as well as in diabetic population which is also in concordance with our results [39, 40]. It has recently been reenforced by a 5-year prospective study by Chow et al. (2007) who found hypoadiponectinemia to be a good predictor of hypertension development even after adjustment for risk factors such as BMI, sex, and age [41]. The inverse correlation between adiponectin concentration and waist circumference was previously described in several studies on MS [8, 13], that is, intra-abdominal fat mass was found to influence the concentration of circulating adiponectin more than subcutaneous fat. Despite whether hypoadiponectinemia is a cause or a consequence of intraabdominal obesity has not been fully addressed yet, so the significantly lower waist circumference and BMI in our group of patients with higher adiponectin concentrations might possibly be explained with an independent adiponectin effect on weight loss. As suggested by Fruebis et al. (2001), adiponectin administration induces weight loss without decreasing food intake in mice consuming a high-fat high-sucrose diet [42].

Additionally, in agreement with adiponectin modulating liver function previosly mentioned, we also found a negative correlation between plasma adiponectin concentrations and hepatic biomarkers, that is, aspartate aminotransferase (AST), alanine aminotransferase (ALT), *γ*-glutamyltransferase (GGT), and alkaline phosphatase (ALP). This is with accordance with the results of López-Bermejo et al. (2004) who reported that adiponectin concentrations were associated with plasma concentrations of various liver function tests in healthy humans suggesting that adiponectin deficiency is an important risk factor for the development of fatty liver, steatohepatitis, and other forms of liver injury [43]. 

The present study has a number of potential limitations. First, our study was cross-sectional, which limited our ability to infer a causal relation between adiponectin concentrations and development of MS in T1DM. Second, we measured insulin sensitivity using clinical parameters with eGDR and did not have access to direct, detailed measures of insulin resistance using euglycemic-hyperinsulinemic clamp test. Third, our analyses were based on a single measurement of total fasting adiponectin that may not reflect the relation over time.

In conclusion, our results highlight the importance of the relationship between adiponectin, IR, and the presence of the MS in T1DM. Whether adiponectin is a marker of MS as well as all components of MS according to the IDF criteria in T1DM patients and whether higher adiponectin concentrations have a protective role in the development of the MS in T1DM need to be clarified in future follow-up studies. 

## Figures and Tables

**Table 1:** Demographic, anthropometric, and clinical characteristics of the 77 patients with type 1 diabetes.

Variable	Mean/median	SD/range
Age (years)	45.66	11.87
Gender (m/f)	47/30	
Duration of diabetes (years)	21.0	1.0–47.0
Weight (kg)	76	52–112
BMI (kg/m^2^)	25	18–36
Waist circumference (cm)	85.6	11.8
HbA1c (%)	7.3	5.4–12.2
SBP (mmHg)	130	100–165
DBP (mmHg)	80	60–110
eGDR (mg/kg^−1^ min^−1^)	6.77	3.78–11.65
LDL cholesterol (mmol/L)	2.95	1.49–5.32
Total HDL cholesterol (mmol/L)	1.56	0.78–3.83
HDL2 cholesterol (mmol/L)	0.44	0.2–2.8
HDL3 cholesterol (mmol/L)	1.13	0.39–2.28
Triglycerides (mmol/L)	1.19	0.61
AST (U/L)	23.5	13–57
ALT (U/L)	24	12–95
GGT (U/L)	20	10–92
ALP (U/L)	76	18–155
Adiponectin (*μ*g/mL)	14.10	3.4–31.4

ALP: alkaline phosphatase, ALT: alanine aminotransferase, AST: aspartate aminotransferase, GGT: *γ*-glutamyltransferase, BMI: body mass index, DBP: diastolic blood pressure, HbA1c: glycated hemoglobin, eGDR: estimated glucose disposal rate, and SPB: systolic blood pressure.

**Table 2:** Clinical and metabolic characteristics of patients depending on adiponectin concentrations.

Variable	Adiponectin < 14.10 *μ*g/mL	Adiponectin > 14.10 *μ*g/mL	*P*
Age (years)	43.8 ± 13.72	46.26 ± 9.79	NS
Gender (m/f)	31/7	16/23	NS
Duration of diabetes (years)	17.5 (1–41)	25 (2–47)	0.015
Weight (kg)	83 (52–112)	68 (54–103)	<0.001
BMI (kg/m^2^)	26 (18–33)	24 (20–36)	0.027
Waist circumference (cm)	90.5 ± 10.4	80.9 ± 11.3	<0.002
HbA1c (%)	7.3 (5.41–11.1)	7.2 (5.4–12.2)	NS
SBP (mmHg)	130 (110–160)	125 (100–165)	NS
DBP (mmHg)	80 (60–110)	80 (60–100)	NS
eGDR (mg/kg^−1^ min^−1^)	6.06 (3.79–10.01)	7.67 (3.78–11.65)	0.003
LDL cholesterol (mmol/L)	2.79 (1.83–5.20)	2.74 (1.49–5.32)	NS
Total HDL cholesterol (mmol/L)	1.46 (0.83–3.83)	1.66 (0.78–2.62)	NS
HDL2 cholesterol (mmol/L)	0.43 (0.20–2.80)	0.44 (0.21–1.05)	NS
HDL3 cholesterol (mmol/L)	1.06 (0.56–1.53)	1.23 (0.39–2.28)	0.011
Triglycerides (mmol/L)	1.27 ± 0.64	1.11 ± 0.58	NS
AST (U/L)	24 (15–42)	22 (13–57)	NS
ALT (U/L)	27.5 (12–91)	22 (12–95)	NS
GGT (U/L)	25 (13–92)	16 (10–59)	0.042
ALP (U/L)	77 (18–155)	75 (36–140)	NS
MS presence (*n*, %)	17 (44.7%)	9 (23.1%)	0.045

ALP: alkaline phosphatase, ALT: alanine aminotransferase, AST: aspartate aminotransferase, GGT: *γ*-glutamyltransferase, BMI: body mass index, DBP: diastolic blood pressure, HbA1c: glycated hemoglobin, eGDR: estimated glucose disposal rate, and SPB: systolic blood pressure.

**Table 3:** Correlation analysis of adiponectin concentrations with metabolic and anthropometric variables.

Variable	
Duration of diabetes (years)	0.370*
Weight (kg)	−0.502*
BMI (kg/m^2^)	−0.367*
Waist circumference (cm)	−0.512*
HbA1c (%)	0.076
SBP (mmHg)	−0.238*
DBP (mmHg)	−0.149
eGDR (mg/kg^−1^ min^−1^)	0.332*
LDL cholesterol (mmol/L)	−0.143
Total HDL cholesterol (mmol/L)	0.332*
HDL2 cholesterol (mmol/L)	0.049
HDL3 cholesterol (mmol/L)	0.222*
Triglycerides (mmol/L)	−0.129
AST (U/L)	−0.062
ALT (U/L)	−0.107
GGT (U/L)	−0.199
ALP (U/L)	−0.34

*P < 0.05.

ALP: alkaline phosphatase, ALT: alanine aminotransferase, AST: aspartate aminotransferase, GGT: *γ*-glutamyltransferase, BMI: body mass index, DBP: diastolic blood pressure, HbA1c: glycated hemoglobin, eGDR: estimated glucose disposal rate, and SPB: systolic blood pressure.

**Table 4:** Logistic regression analysis of adiponectin concentrations and diabetes duration with development of metabolic syndrome in type 1 diabetic patients according to the IDF criteria.

Independent variable	Model A	Model B	Model C
(a) Adiponectin	0.91 (0.84–0.98)*	0.89 (0.82–0.97)*	0.89 (0.81–0.97)*

Data are OR (95% CI) from separate models. Model A crude; model B adjusted for age and sex, model C adjusted for age, sex, duration of diabetes, and the use of statins.

*P < 0.05.
